# A Comparison of Cardiometabolic Risk Factors in Households in Rural Uganda With and Without a Resident With Type 2 Diabetes, 2012–2013

**DOI:** 10.5888/pcd12.140486

**Published:** 2015-04-02

**Authors:** Jannie Nielsen, Silver K. Bahendeka, Edward W. Gregg, Susan R. Whyte, Ib C. Bygbjerg, Dan W. Meyrowitsch

**Affiliations:** Author Affiliations: Silver K. Bahendeka, St Francis Hospital Nsambya, Kampala, Uganda; Edward W. Gregg, Division of Diabetes Translation, Centers for Disease Control and Prevention, Atlanta, Georgia, USA; Susan R. Whyte, Department of Anthropology, University of Copenhagen, Copenhagen, Denmark; Ib C. Bygbjerg, Dan W. Meyrowitsch, Global Health Section, Department of Public Health, University of Copenhagen, Copenhagen, Denmark.

## Abstract

**Introduction:**

Few studies have examined the health consequences of living in a household with a person who has been diagnosed with type 2 diabetes (T2D). We assessed the association of sharing a household with a person with diagnosed T2D and risk factors for cardio-metabolic diseases in Uganda, a low-income country.

**Methods:**

Ninety households with 437 residents in southwestern Uganda were studied from December 2012 through March 2013. Forty-five of the households had a member with diagnosed T2D (hereafter “diabetic household”), and 45 households had no member with diagnosed T2D (hereafter “nondiabetic household”). We compared glycosylated hemoglobin (HbA1c), fasting plasma glucose (FPG), hypertension, anthropometry, aerobic capacity, physical activity, nutrition, smoking, and diabetes-related knowledge of people without diagnosed T2D living in diabetic and nondiabetic households.

**Results:**

People living in diabetic households had a significantly higher level of diabetes-related knowledge, lower levels of FPG (5.6 mmol/L vs 6.0 mmol/L), and fewer smoked (1.3% vs 12.9%) than residents of nondiabetic households. HbA1c was significantly lower in people aged 30 years or younger (5.2% vs 5.4%) and in males (5.2% vs 5.4%) living in diabetic households compared to residents of nondiabetic households. No differences were found between the 2 types of households in overweight and obesity, upper-arm fat area, intake of staple foods or cooking oil, or physical activity.

**Conclusions:**

Sharing a household with a person with T2D may have unexpected benefits on the risk factor profile for cardio-metabolic diseases, probably because of improved health behaviors and a closer connection with the health care system. Thus, future studies should consider the household for interventions targeting primary and secondary prevention of T2D.

## Introduction

In 2013, an estimated 382 million adults worldwide were living with diabetes, and projections suggest that more than 592 million adults will have the disease in 2035 (1). Although the reported prevalence of diabetes in Sub-Saharan Africa is low (3.8%), approximately 75% of all people with diabetes are unaware they have the disease and therefore receive no treatment (2). Moreover, the largest proportional increase in prevalence is expected to occur in Sub-Saharan Africa (1). In Uganda, prevalence of diabetes in adults has been estimated at 7.4% in rural areas (3) and 8.1% in urban areas (4). This situation challenges a country already heavily burdened with communicable diseases, financial constraints, and limited medical resources for diabetes care (5,6).

Lifestyle-related factors play an important role in the development of T2D (7,8), and studies from both middle- (9,10) and high-income countries (11,12) show that T2D can be prevented or delayed through a balanced diet, increased physical activity, and weight loss. However, it remains unclear how this evidence should be translated into effective and feasible population-wide interventions in low-, middle-, and high-income countries.

In many low-income countries like Uganda, daily life is focused around the family and the household. In households in which 1 member has T2D, that member may affect household knowledge and lifestyle practices and thus influence cardio-metabolic risk for other household members. The genetic inheritance of T2D is clearly established (13,14), but few studies have examined the effect of household sharing on the risk factors for T2D and related cardio-metabolic conditions of people without T2D (13,15–18).

Because the household environment in rural Uganda could be an important factor in either increasing risk or decreasing risk for chronic diseases, we compared the cardio-metabolic risk factors of people without diagnosed diabetes who live in diabetic households with those who live in nondiabetic households.

## Methods

This cross-sectional study comprised 90 households of which half included a person with diagnosed T2D. All households were within the Kagando Hospital catchment area (defined as within a travel time of 15 minutes by walking to 1 hour by vehicle to the hospital). Kagando Hospital is a rural, private, not-for-profit health facility located in Kasese District in southwestern Uganda. The Kasese population is young (57.5% aged 18 years or younger) (19). Households comprise 1 to 3 generations. The fertility rate is high (7.4%) (20), and polygamy is common. The district is mountainous, and 75.3% of the inhabitants live in rural areas; small-scale farming of cassava, plantain, mango, and cash crops such as coffee is the main occupation (20). The majority of people live in houses made of mud or sun-dried bricks with an iron sheet roof, no electricity, and no piped water. Kagando Hospital serves a population of 400,000 and operates a weekly diabetes clinic with diabetes education focused on medication, diet, physical activity, and smoking cessation.

A household was defined as a place where people live together and share food on a daily basis. Households with a resident with diabetes were selected from 354 diabetes patients’ records at the Kagando Hospital diabetes clinic, of which 79 fulfilled the following inclusion criteria at the patient level: the patient had lived with a diagnosis of diabetes for at least 2 years, had attended at least 2 diabetes clinic consultations, and was aged 40 years or older at diagnosis. The inclusion criteria for participation at the household level were that the household contained at least 3 members aged 13 years or older and at least 2 generations (children and parents or children and grandparents) lived in the household. Households were excluded if 1 household member had a diagnosis of HIV/AIDS or type 1 diabetes or had active tuberculosis, severe mental illness, alcoholism, or drug addiction. Six diabetic households did not meet these criteria and were excluded from the study.

Nondiabetic households were evaluated by the same inclusion and exclusion criteria as diabetic households with the additional requirement that no household member had received a diagnosis of T2D. We used a random sampling plan to select nondiabetic households. To sample a nondiabetic household, we visited the fifth house to the left of a diabetic household, facing the front of the diabetic household. If the visited household included a person within 5 years of age of the person with T2D in the diabetic household and the household overall met our inclusion criteria, the household was invited to participate in the study. If no one in this age range lived in the household or if the household did not fulfill our inclusion criteria, we visited the next house to the left to find out if this household fulfilled the inclusion criteria. The sampling process continued until a nondiabetic household meeting all the criteria was identified.

Of 100 households invited to participate (45 with a resident with diabetes and 55 without), 90 households with a total of 437 residents agreed to participate (response rate 97.5%). The 45 household residents with diagnosed T2D had attended an average of 21 consultations (range 2–64) at the diabetes clinic over 2 to 9 years, beginning when the hospital began keeping files on the patients at the diabetes clinic. To compare risk factors for cardio-metabolic diseases between households with and without a resident with diagnosed diabetes, the 45 people who already had a diagnosis of diabetes were excluded, yielding a sample of 392 participants without diagnosed diabetes.

Household members were examined in their own home. Informed written consent was obtained from all participants, and the study was approved by the Uganda National Council of Science and Technology, the Makerere University School of Medicine Research and Ethics Committee, St Francis Hospital Nsambya, and Kagando Hospital.

### Measurements

Following an overnight fast, hemoglobin A1c (HbA1c) was measured with an Afinion AS100 Analyzer (Axis Shield PoC, Norway), and fasting plasma glucose (FPG) (mmol/L) was measured with an Accu-check Aviva glucose meter (Roche Diagnostics, Switzerland). Diabetes was defined as HbA1c ≥6.5% (21) or FPG ≥7.0 mmol/l (22), and high risk of diabetes as HbA1c ≥5.7% or FPG ≥6.1 mmol/l.

Blood pressure (BP) was measured with an Omron M6 HEM7211E (Omron Global, Kyoto, Japan) in sitting position after at least 10 minutes of rest and hypertension was defined as the presence of antihypertensive therapy, or a systolic BP at or above 140 mm Hg, or a diastolic BP at or above 90 mm Hg, averaged over the last 2 blood pressure levels (23).

Body weight was measured to the nearest 0.1 kg with the participant wearing light clothes (SECA flat scale model 876, SECA UK), and height was measured to the nearest 0.1 cm (SECA portable stadiometer, model 213, SECA UK). Body mass index (BMI) (weight [kg]/height [m^2^]) was categorized for adults according to the World Health Organization (WHO) classifications (24) and for adolescents aged from 13 to 19 years according to WHO Child Growth Standards (25). Overweight and obese individuals were grouped together because of few observations in the obese group (n = 13).

Waist circumference was measured midway between the iliac crest and the costal margin following a quiet expiration with SECA ergonomic circumference measuring tape 201 (SECA UK) and was used to calculate waist:height ratio (WtHR) (26). On the nondominant arm, mid–upper-arm circumference was measured with SECA ergonomic circumference measuring tape and used with the triceps brachii skinfold thickness (Harpenden Caliper, model RH15-9-LR, Baty International) to calculate upper-arm fat area (UFA) according to Frisancho (27). WtHR and UFA were used as continuous variables.

Aerobic capacity was estimated on the basis of an 8-minute step test with heart rate measurements (Polar RS100) every 30 seconds followed by 2 minutes of rest with heart rate measurements every 15 seconds, and the measurements were managed according to the Cambridge protocol (28). In data analyses, participants who could not perform or complete at least 4 minutes of the step test were coded as unfit with the exception of those who were pregnant, had recently given birth, or had an acute illness.

A modified version of the International Physical Activity Questionnaire (29) was used to assess physical activity level. Socioeconomic status (SES), education, family history of diabetes, and smoking habits were also assessed by questionnaire. A food frequency questionnaire was developed on the basis of observation studies at markets, small shops, cooking sessions in private households, and informal interviews with people in the study setting. This questionnaire determined consumption of cooking oils and the staple foods cassava (root and flour), maize flour, sweet potatoes, potatoes, plantains, sorghum, and rice, all staples that are high-glycemic index foods associated with the development of T2D (30). A questionnaire capturing knowledge about diabetes was developed on the basis of observational studies conducted at the diabetes clinic at Kagando Hospital. The questionnaire score ranged from −32 to 32, with the maximum indicating all questions were correct.

All questionnaires were either developed for or adapted to local cultural patterns and were translated into the local language, Lukonjo, and back-translated into English. All questionnaires went through a pilot study to test participants’ understanding, interpretation, and perception of the questions. Trained local assistants and the first author (J.N.) conducted physical examinations, and local assistants conducted all questionnaires.

### Statistical analysis

Our primary analyses compared the HbA1c; FPG; hypertension; smoking; anthropometric-, diet-, and physical activity-related risk factors; and diabetes-related knowledge of household residents aged 13 years or older in diabetic households who had not previously received a diagnosis of diabetes with those in nondiabetic households.

Key covariates in the analyses were sex, age, pregnancy, SES, and education. Age was calculated on the basis of date of birth. In cases of unknown birth year, we used a locally developed event calendar with major events in the study setting taking place between 1933 and 2002. The participants were then asked to link their birth year and other major biographic milestones to the events in calendar in order to determine the year of birth.

A principal component analysis (31) of employment, land size, housing condition, value of domestic animals, education level of the head of household, and value of household possessions was performed to determine SES at the household level, and all residents within the same household were assigned the same SES.

A general mixed model was used to model outcome variables as a function of living in a household with a member with diagnosed T2D. Household was used as a random effect to account for within-household clustering. Differences in categorical variables were tested by using ordinal logistic regression adjusted for confounding and clustering at the household level. We also tested for interactions with the main outcome variables and the interaction between household and age and sex, respectively; we grouped residents into 2 age groups (<30 y and ≥30 y). A univariate model was used to test the association between diabetes-related knowledge and different cardio-metabolic risk factors. All continuous variables were transformed in the statistical analyses; means were then back-transformed by using the geometric mean. Thus, results are presented as mean values and interquartile range (25th and 75th percentiles) for households with and without a resident with a diagnosis of diabetes.

## Results

### Characteristics of participants in diabetic and nondiabetic households

Overall, SES and educational level were higher in diabetic households than in nondiabetic households (*P* = .001 vs *P* = .003), but no differences in sex or age existed between the 2 types of households (*P* = .27 and *P* = .92). To compare risk factors for cardio-metabolic diseases between diabetic households and nondiabetic households, the 45 persons with diagnosed T2D were excluded, yielding a final sample of 392 participants. Of those without diagnosed diabetes, 27 met the criteria for diabetes, and 131 were at high risk of diabetes. The number of participants with undiagnosed diabetes or at high risk for diabetes did not differ significantly between household types, and their data were not excluded from the further analysis. After exclusion of the 45 participants with diagnosed T2D, residents of diabetic households differed from nondiabetic households (Table 1) in that they were younger (median age 23.8 y for diabetic household vs 31.5 y for nondiabetic households, *P* = .006), had more women (66.9% vs 54.7%, *P* = .016), had higher SES (*P* = .001), and had a higher prevalence of residents with a positive family history of diabetes (65.0% vs 3.5%, *P* < .001).

**Table 1:** Background Characteristics of Residents of Diabetic Households and Nondiabetic Households^a^ Study of Cardio-Metabolic Risk Factors in Households in Rural Uganda, 2012–2013

Demographic Characteristics	Total (n = 392)^b^	Residents of Diabetic Households (n = 160)^b^	Residents of Nondiabetic Households (n = 232)^b^	Crude *P* Values
**Male**	158 (40.3)	53 (33.1)	105 (45.3)	.016^c^
**Female**	234 (59.7)	107 (66.9)	127 (54.7)	
**No. of household residents aged ≥13 y^d^**	5 (3–10)	4 (3–9)	5 (3–10)	.02^e^
**Median age of household residents, y^e^**	26.6 (18.1;51.8)	23.8 (17.3;46.0)	31.5 (18.5;54.8)	.006^f^
**Level of education**				
Did not complete primary school	264 (67.3)	99 (61.9)	165 (71.1)	.06
Completed primary school	109 (27.8)	48 (30.0)	61 (26.3)	
Completed secondary school	19 (4.8)	13 (8.1)	6 (2.6)	
**Family history of diabetes n (%)^g^**	112 (28.6)	104 (65.0)	8 (3.5)	<.001
**Socioeconomic status at household level (n = 90)**				
Low	30 (33.3)	10 (22.2)	20 (44.4)	.001^c^
Middle	30 (33.3)	12 (26.7)	18 (40.0)	
High	30 (33.3)	23 (51.1)	7 (15.6)	

a A diabetic household is one in which 1 household resident has received a diagnosis of type 2 diabetes; a nondiabetic household is one in which no resident has received a diagnosis of type 2 diabetes.

b Values are stated as n (%) unless otherwise indicated.

c Calculated by χ^2^ test.

d Median (range).

e Median (25th; 75th percentiles).

f Wilson rank–sum test.

g One value was missing for 1 participant from a nondiabetic household.

### Cardio-metabolic risk factors

Crude and adjusted analyses showed that FPG levels and prevalence of smokers were significantly lower in diabetic households (0.4 mmol/L and 11.6 percentage points lower than in nondiabetic households) (Table 2). The level of diabetes-related knowledge was significantly higher in diabetic households than in nondiabetic households (*P* < .01 in all tests). Prevalence of underweight was lower in diabetic households than in nondiabetic households (3.1% vs 12.5%, *P* = .006); however, there was no significant difference in the prevalence of overweight and obesity (19.4% vs 13.8%, *P* = .32). After adjusting for age, sex, and pregnancy, the levels of HbA1c, hypertension, upper-arm fat area (UFA), waist-to-hip ratio (WtHR), physical activity level, estimated aerobic capacity, weekly servings of staple foods, and intake of cooking oil did not differ significantly between the 2 types of households (*P* > .05 for all tests). Including SES and education as covariates in the general mixed model did not change our findings (Table 2).

**Table 2:** Cardio-Metabolic Risk Factors Among Residents (N = 392) of Diabetic Households and Nondiabetic Households,^a^ Study of Cardio-Metabolic Risk Factors in Households in Rural Uganda, 2012–2013

Risk Factor	Residents of Diabetic Households,^b^ n = 160	Residents of Nondiabetic Households,^b^ n = 232	Crude *P* Value	Adjusted *P* Value^c^
HbA1c, %^d^	5.4 (5.1;5.6)	5.4 (5.2;5.7)	.14	.17/.15
FPG, mmol/L^d^^,^ e	5.6 (5.2;6.1)	6.0 (5.5;6.3)	.001	.002/.01
Hypertension^f^	37 (23.4%)	60 (26.1%)	.57	.11/.09
BMI				
Underweight	5 (3.1)	29 (12.5)	.009	.03/.29
Normal weight	124 (77.5)	171 (73.7)		
Overweight or obese	31 (19.4)	32 (13.8)		
Upper-arm fat area, cm^2,^ d	12.9 (9.1;19.4)	11.4 (7.7;18.7)	.08	.47/.56
Waist-height ratio d ^,^ g	0.48 (0.44;0.51)	0.47 (0.44;0.50)	.48	.35/.77
Estimated aerobic capacity^h^				
Very low	12 (7.6)	35 (15.3)	.32	.72/.80
Low	34 (21.5)	50 (21.8)		
Medium	87 (55.1)	105 (45.9)		
High	25 (15.8)	39 (17.0)		
Physical activity level^i^				
Low	3 (1.9)	11 (4.7)	.573	.59/.86
Moderate	33 (20.9)	46 (19.8)		
High	122 (77.2)	175 (75.4)		
Staple foods, servings per week^d^	26.3 (21;34)	28.4 (24;36)	.71	.71/.45
Cooking oil, high intake	66 (41.3)	134 (57.8)	.14	.11/.035
Smoking status				
Never smoked	147 (91.9)	171 (73.1)	<.001	.002/.008
Former smoker	11 (6.9)	31 (13.4)		
Smoker	2 (1.3)	30 (12.9)		
Knowledge diabetes score^j^	8.3 (−6,18)	6.4 (−6,17)	<.001	<.001/.002

a A diabetic household is one in which 1 resident has received a diagnosis of type 2 diabetes; a nondiabetic household is one in which no resident has received a diagnosis of type 2 diabetes. Pregnant women were not excluded from the comparison of risk factors between the 2 types of household; however, excluding the pregnant women from the comparison would not change the risk-factor association between exposure and outcome in any of the analyses.

b Values are stated as n (%) unless otherwise indicated.

c *P* values are adjusted for sex, age, and pregnancy and for sex, age, pregnancy, education, and SES.

d Data are means (25th;75th percentiles).

e During the examinations 21 participants did not report they were fasting and were therefore excluded from the analysis of FPG.

f Data missing on 4 individuals; one female had an upper-arm circumference too large for the BP cuff and 3 participants had values out of the measuring range of the BP device.

g Data is missing on one individual because no waist measurement was made.

h Data are grouped according to Astrand (36); data are missing on 5 individuals because of pregnancy, recent delivery, sickness, or technical error.

i Physical activity data are used in International Physical Activity Questionnaire truncated format (37); data are missing on 2 individuals.

j Data are means (range); higher score corresponds to higher diabetes-related knowledge.

### Stratification of risk factors by age and sex

We observed a significant interaction between sex and household diabetes status (*P* = .026) and between age and household diabetes status (*P* = .008) in relation to HbA1c levels. We also found a significant interaction between age and household diabetes status in body mass index (BMI), WtHR, and smoking (*P* < .05 for all tests). No interactions between age or sex and household diabetes status were observed for any other outcome variables (*P* > .10 for all tests).

In age-stratified analyses, HbA1c was significantly lower in the younger age group (household members below 30 years of age) in diabetic households than in nondiabetic households, both before and after adjustment for confounding (5.2% vs 5.4%, *P* = .006), but no difference was found in the older age group (5.6% vs 5.5%, *P* = .31). BMI was significantly different in the older age groups from the 2 types of households (*P* = .007): fewer residents were underweight in diabetic households than in nondiabetic households (4.9% vs 21.2%, *P* = .007). WtHR was significantly higher among older adults in diabetic households than in nondiabetic households (0.51 vs 0.48, *P* = .006). The older age group in diabetic households also included a lower proportion of former and current smokers (13.1% vs 22.9%, former smokers; 0.0% vs 22.9%, current smokers, *P* < .001).

In sex-stratified analyses, the level of HbA1c was significantly lower among males in diabetic households than among males in nondiabetic households (5.2% vs 5.4%, *P* = .019), whereas there was no difference between females from the 2 types of households (5.4% vs 5.5%, *P* = .99).

### Association between knowledge and cardio-metabolic risk factors

No associations between diabetes-related knowledge and the different risk factors for cardio-metabolic diseases were found (*P* > .10 for all tests). Including diabetes-related knowledge as a covariate in the analyses of HbA1c, FPG, and smoking did not considerably diminish the difference in these variables between the 2 types of households.

## Discussion

Our results suggest that residing in a household with a person with T2D is associated with a more favorable cardio-metabolic risk factor profile (including lower levels of blood glucose and smoking, and a higher level of diabetes-related knowledge) than residing in a nondiabetic household. These findings were most pronounced among younger people (13– <30 y), where HbA1c was lower in diabetic households. Among older people (30–92.5 y), smoking prevalence was lower in diabetic households. Furthermore, males in diabetic households had lower HbA1c.

The magnitude of difference in smoking prevalence (11.6 percentage points) is likely to be associated with important differences in risk for diabetes, heart disease, and pulmonary disease (32). This potential advantage in cardio-metabolic risk profile is further strengthened by the 0.44 mmol/L difference in FPG observed between diabetic and nondiabetic households. A longitudinal study from South Africa found that people progressing from no diabetes to diabetes had FPG 0.30 mmol/L higher than those who did not progress to diabetes (33). On the other hand, the same South African study (33) found that people progressing from no diabetes to diabetes had HbA1c% 0.30 higherthan those not progressing to diabetes, which is slightly higher than the 0.15 to 0.21 difference in HbA1c% observed among young individuals and males in our study.

The favorable cardio-metabolic risk factor profile is also consistent with US studies that found greater weight loss and decreased energy intake in untreated spouses of people participating in lifestyle interventions (16,18). In contrast, another US study found that relatives of a person with T2D were more obese, despite having a higher awareness of diabetes risk factors, than those without a family history of diabetes (34). However, the relatives in that study (34) did not share a household and daily life with the person with T2D as in our study and the published intervention studies (16,18).

The higher level of diabetes-related knowledge and education in the diabetic households may play a role in the decreased risk factor levels shown in the present study. However, we observed no association between diabetes-related knowledge and risk factors for cardio-metabolic diseases. Furthermore, including diabetes-related knowledge as a covariate in the analyses of HbA1c, FPG, and smoking did not considerably diminish the difference in these variables between the 2 types of households. The lack of an association between knowledge and improved cardio-metabolic risk factor profile is consistent with an evaluation study by Yoder et al, which found no clear relationship between changes in knowledge and changes in behaviors (35).

Thus, it remains possible that habits, behaviors, cooking practices, and social pressure favoring exercise and nonsmoking affect health behaviors in residents of diabetic households without affecting diabetes knowledge per se. Therefore, our results and the existing literature suggest that diabetes-related education or T2D in the family alone are not sufficient to facilitate a change in practice. Instead, it may be the combination of education and sharing daily life that is important. Future studies should examine whether this combination of factors is important in other settings, such as in urban Sub-Saharan Africa or other low-income countries.

Smoking cessation was also recommended at the diabetes clinic, and 12 of the people with diagnosed T2D reported they were former smokers (results not reported because we excluded people with diagnosed T2D). These people’s smoking cessation may have affected other members of the diabetic household and thereby explains the lower number of smokers in these households.

Diabetic households had a higher SES, which could have increased the intake of more expensive food items not produced by the household itself, such as cooking oil. However, we found no difference in cooking oil use between the 2 types of households, although 3 diabetic households reported not using oil at all. We observed no effect of SES or education adjustment on the association between type of household and diabetes-related knowledge.

Our study had some limitations. First, the cross-sectional design prevents confirmation that diabetes status caused the favorable risk factor status in diabetic households, because cardio-metabolic risk factor status before the person’s diabetes diagnosis is unknown. However, given that 1 person in each diabetic household had T2D, it is unlikely that diet was healthier and physical activity levels were higher in diabetic households before the person was diagnosed with diabetes.

Nutritional data were estimated by using a household-level variable that assumed that everyone aged 13 years or older consumed the same number of servings, which may not be the case. Finally, the people with T2D were selected from a private not-for-profit hospital and had attended a minimum of 2 consultations at the diabetes clinic. Therefore, these people may have higher SES and may not be representative of Ugandans with T2D in general.

In conclusion, this study found that young residents (13–<30 y) of a diabetic household were at lower risk for cardio-metabolic diseases than their counterparts from nondiabetic households. Furthermore, people aged 30 years or older from diabetic households were not at higher risk than the same age group from nondiabetic households despite their shared daily life, environment, and perhaps even genes with a person with diagnosed T2D. We are not aware of other studies reporting a more favorable cardio-metabolic risk factor profile in residents of diabetic households than those living in a nondiabetic household, whether from Africa or from other parts of the world where diabetes has been much more researched. The results suggest that people benefit from sharing a household with a person with T2D who has received diabetes-related education. The potential cardio-metabolic benefits may occur as unintended spill-over effects. Thus, diabetic households may be a potential avenue for primary prevention of T2D in countries with limited resources for addressing the increasing burden of diabetes. Further research needs to focus on exploiting the potential of the household — both households with and without a chronic disease — for health promotion in general and prevention of cardio-metabolic diseases specifically.
